# Patterns of Fish Connectivity between a Marine Protected Area and Surrounding Fished Areas

**DOI:** 10.1371/journal.pone.0167441

**Published:** 2016-12-01

**Authors:** Rita Sahyoun, Paolo Guidetti, Antonio Di Franco, Serge Planes

**Affiliations:** 1 EPHE, PSL Research University, UPVD, CNRS, USR 3278 CRIOBE, Perpignan, France; 2 Université Côte d'Azur, UCA, CNRS, ECOMERS, Parc Valrose, Avenue Valrose, Nice, France; 3 CoNISMa—Consorzio Nazionale Interuniversitario per le Scienze del Mare, Piazzale Flaminio, Rome, Italy; University of California Santa Cruz, UNITED STATES

## Abstract

Patterns of connectivity and self-recruitment are recognized as key factors shaping the dynamics of marine populations. Connectivity is also essential for maintaining and restoring natural ecological processes with genetic diversity contributing to the adaptation and persistence of any species in the face of global disturbances. Estimates of connectivity are crucial to inform the design of both marine protected areas (MPAs) and MPA networks. Among several approaches, genetic structure is frequently used as a proxy for patterns of connectivity. Using 8 microsatellite loci, we investigated genetic structure of the two-banded sea bream *Diplodus vulgaris*, a coastal fish that is both commercially and ecologically important. Adults were sampled in 7 locations (stretches of coastline approximately 8 km long) and juveniles in 14 sites (~100 to 200 m of coastline) along 200 km of the Apulian Adriatic coast (SW Adriatic Sea), within and outside an MPA (Torre Guaceto MPA, Italy). Our study found similar genetic diversity indices for both the MPA and the surrounding fished areas. An overall lack of genetic structure among samples suggests high gene flow (i.e. connectivity) across a scale of at least 200 km. However, some local genetic divergences found in two locations demonstrate some heterogeneity in processes renewing the population along the Apulian Adriatic coast. Furthermore, two sites appeared genetically divergent, reinforcing our observations within the genetic makeup of adults and confirming heterogeneity in early stage genetics that can come from either different supply populations or from chaotic genetic patchiness occurring under temporal variation in recruitment and in the reproductive success. While the specific role of the MPA is not entirely known in this case, these results confirm the presence of regional processes and the key role of connectivity in maintaining the local population supply.

## Introduction

Levels of self-recruitment within and connectivity among populations are key factors influencing marine population persistence and stock sustainability [1], as well as the efficiency of management strategies such as marine protected areas (MPAs). MPAs are increasingly being implemented as tools to simultaneously achieve both fisheries management and biodiversity conservation objectives [2–7]. Significant levels of self-recruitment may be particularly important for single, isolated MPAs for which there are no other reliable sources of larvae [8]. Plus, understanding the rate of exchange among populations will help define the spatial scale at which the implementation of future MPAs and MPA networks would be effective [9–13]. Specifically, the functioning of an MPA is dependent on the degree to which individuals inhabiting the MPA contribute to populations within the MPA [9, 14] and to spillover/dispersal beyond their boundaries [15, 16]. Connectivity thus influences the extent to which MPAs may contribute recruits to surrounding fished areas, as well as to other MPAs [17].

Population genetics has been and remain the most widely used approach to indirectly evaluate connectivity from genetic structure (i.e. heterogeneity) among populations in marine organisms [18, 19] assuming several limits in the interpretation [20]. Genetic tools are also a powerful resource to describe population dynamics and to predict, validate and quantify the ecological and economical success of MPAs [21]. Among the numerous molecular markers available for these types of studies [22] microsatellites are usually used because of their high polymorphism [23] enabling them to detect fine-scale genetic differentiation between samples [24, 25].

In this study, we investigated patterns of connectivity between an MPA and surrounding fished areas using the two-banded sea bream *Diplodus vulgaris* (Geoffroy Saint-Hilaire, 1817) as a model species and Torre Guaceto (hereinafter TGMPA, SW Adriatic Sea) as a model MPA. We selected the two-banded sea bream because of its positive response to MPA in terms of biomass recovery [26, 27] and its ecological importance in the Mediterranean Sea, being the major predator of sea urchins and playing a major role in controlling their abundance and effects on benthic communities [28–30]. This species has also a socio-economic value and supports local artisanal and recreational fisheries [29, 31]. It has a pelagic larval duration ranging from 25 to 61 days in the Adriatic Sea [32] and adults have sedentary behavior with home range size less than 1 km^2^ [33, 34]. Limited information was available concerning its genetic structure. A single genetic study using allozyme markers revealed a genetic substructure among the samples examined within the east Mediterranean basin [35]. Galarza et al. [36] showed significant differences in the estimates of genetic divergence between *D*. *vulgaris* populations across the Almeria-Oran front in the western Mediterranean. Whereas, other studies conducted on its congeneric species *D*. *sargus* showed weak but significant genetic differences for populations at a scale ranging from less than 100 km [37] to several hundreds of kilometers [38].

The TGMPA (SW Adriatic Sea) was adopted as a model MPA because of its effective enforcement since 2001 and because previous studies showed that fishing bans within its no-take zones allowed for the recovery of both fish [39, 40] and benthos [29]. TGMPA covers 2227 ha, stretching along about 8 km of coastline, and is subdivided into three zones: (1) a no-take/no-access reserve (zone A, 179 ha); (2) a general reserve (zone B, 163 ha) and (3) a partial reserve (zone C, 1885 ha), where restrictions to human activities become progressively less severe. Access to zone A is restricted to scientists, reserve personnel and police authorities. In zone B only recreational bathing from the coast is allowed. In zone C, both professional and recreational fishing are allowed subject to permission of TGMPA management body, with the exception of spearfishing. Here, we used microsatellite DNA markers to 1) study the genetic structure of *D*. *vulgaris* along about 200 km of coast encompassing an MPA and fished areas, and 2) to estimate the amount of immigrants and self-recruitment in TGMPA, using assignment tests [41–43].

## Material and Methods

### Sampling and genotyping

**Fig 1:**
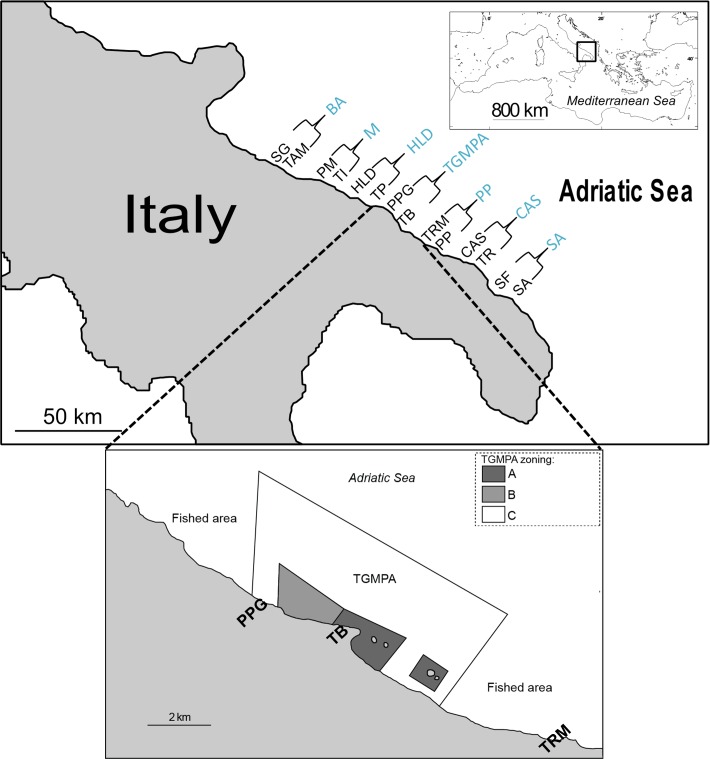
Study area with sampling locations and sites: Locations: BA = Bari, M = Monopoli, HLD = Hotel La Darsena, TGMPA = Torre Guaceto MPA, PP = Punta Penna, CAS = Casalabate, SA = San Andrea. Sites: SG = San Giorgio, TAM = Torre A Mare, PM = Porto Marzano, TI = Torre Incina, HLD = Hotel La Darsena, TP = Torre Pozzella, PPG = Punta Penna Grossa, TB = Terza Baia, TRM = Torre Rossa Mossa, PP = Punta Penne, CAS = Casalabate, TR = Torre Rinalda, SF = San Foca, SA = San Andrea. “Public domain source of backgrounds maps: OpenStreetMap contributors, available under ODbL licence at http://www.openstreetmap.org/”.

To assess the observed genetic differentiation at a local scale, 7 locations (i.e. stretches of coastline approximately 8 km long) were selected starting from TGMPA, spanning both northward and southward, up to about 100 km from the MPA borders (Fig 1).

Overall, 525 adults from 7 locations were sampled from April 2010 to July 2011 (S1 Table). In conjunction with the adult sampling efforts, a total of 755 juveniles (S2 Table) were sampled in May 2010 at the same 7 locations where the adults were sampled but duplicating sampling in each location (i.e. randomly selecting two sites within each location; Fig 1) to account for potential local variability. The experimental fishing protocol, were carried out at all locations in strict accordance with authorization protocols provided by the Italian Ministry of Agriculture, Foods and Forestry Politics (Permit Number: 0011267–2010). For fishing at the two sites located within the TGMPA, the MPA management body provided a specific authorization (number 0003583-PM-11). After collection, individuals were immediately euthanized in an ice slurry (<5°C) and then immersed in alcohol, following Directive 2010/63/EU of the European Parliament and of the Council on the protection of animals used for scientific purposes. The sampling activity did not involve endangered or protected species. Fin clip of adults was preserved in 95% ethanol for further DNA extraction.

Microsatellite loci were optimized from Roques *et al*. [44, 45] and new primers were developed since several loci showed significant departures from the Hardy-Weinberg equilibrium (HWE) as well as a deficit of heterozygosity due the presence of null alleles (S3 Table). A total of 8 microsatellite loci were screened for all adults and juveniles (S3 Table).

### Genetic statistical analysis

Traditional genetic statistics were performed using F_STAT_ v. 2.9.3 [46] to describe the variability of the 8 microsatellites and an analysis of variance (ANOVA) was performed to test for heterogeneity in genetic diversity indices among adult and juvenile samples. Potential departures from HWE were examined through exact tests with significance determined by a Markov Chain randomization (10,000 dememorizations, 10,000 batches and 5,000 iterations per batch) using GENEPOP 3.4 [47].

The data were tested for the presence of null-alleles using MICROCHECKER v. 2.2.3 [48]. Deviations from HWE and linkage disequilibrium (LD) between loci in each sample were tested in GENEPOP, adjusting for multiple comparisons using a sequential Bonferroni correction [49].

### Population genetic structure

F_STATISTICS_ were computed through an analysis of molecular variance (AMOVA) performed in GENALEX v. 6.502 [50, 51]. Genotypes of juveniles and adults were analyzed separately and later pooled together. Tests for statistical significance for all estimates were based on 10,000 random permutations and significance levels were adjusted using sequential Bonferroni correction [49] for multiple tests. Genetic differentiation estimates (Dest) [52] were also conducted in GENALEX v. 6.502 and the results are in the supporting information (S6 and S7 Tables).

Pairwise *F*st measures were graphically represented through multidimensional scaling (MDS) using PRIMER 5 v.5.2.9. This method computes coordinates for each sample such as the distance between points fitted to the measured distances between populations. The accuracy of the data was measured with a stress factor [53].

In parallel, STRUCTURE 2.3.1 [42, 54, 55] was used to disentangle the total variance within the global data into divergent groups of homogeneous genotypes. Separate analyses were conducted for adults and juveniles followed by a third analysis where all samples were mixed together (adults and juveniles) to examine how juveniles segregate respect to adults. The length burning period was set at 10,000 replications and the Markov Chain Monte Carlo (MCMC) steps at 40,000 as proposed by Pritchard *et al*. [42]. Four runs were carried out for each data set (adults, juveniles and adults + juveniles). Best estimates of K were inferred using the methods of Evanno et al. [56] as implemented in STRUCTURE Harvester [57].

### Assignment tests

Juveniles were assigned to the 7 putative adult samples based on their multi-locus genotype probabilities using a Bayesian assignment method [41]. This method was used with probability estimates generated by 10,000 iterations of Monte Carlo re-sampling using a published algorithm [43] in GENECLASS v.2.0. [58]. Juveniles were considered immigrants when the probability of being assigned to any sample was lower than 0.05 (type I error). When a juvenile showed probabilities of assignment greater than 0.05 to only one sample it was assigned to that sample. Finally, when a juvenile was assigned to more than one adult sample (with *P* > 0.5) it was left as unassigned.

## Results

### Adult population’s genetic diversity and structure

All loci appeared to be moderately to highly polymorphic, exhibiting 7–28 alleles per locus (S1 Table). Allelic richness (Ar) ranged from 6 to 24.89. He and Ho ranged from 0.53 to 0.94 and from 0.54 to 0.94, respectively (S3 Table). All loci were considered statistically independent since no evidence of linkage disequilibrium (LD) was found between any pair of loci while applying a sequential Bonferroni correction. No homozygosity for null alleles was observed and tests did reveal a null allele. However, some of the loci showed a deficit in heterozygosity in some samples (*F*is, S3 Table) probably due to a Wahlund effect by which the presence of different genetic stocks in a single sample can cause an excess of homozygotes.

Genetic diversity indices appeared quite homogeneous among adult samples (S1 Table) with no significant differences found among them using a one-way ANOVA (*P* > 0.5). Allelic richness (Ar) ranged from 13.9 to 15.27, while He and Ho ranged from 0.79 to 0.81 and from 0.70 to 0.75, respectively.

The AMOVA analysis showed that only 1% of the genetic variation was due to the segregation among the 7 adult samples with an overall *F*st equal to 0.006 (*P* < 0.001). Levels of differentiation, as measured by pairwise *F*st, between pairs of samples were low (Table 1).

**Table 1:** Matrix of pairwise *F*st between adult samples. See Fig 1 for legends.

	OUT	OUT	MPA	OUT	OUT	OUT	OUT
	BA	M	HLD	TGMPA	PP	CAS	SA
**BA**	0						
**M**	**0.0256**	0					
**HLD**	0.0052	**0.0140**	0				
**TGMPA**	**0.0084**	0.0091	0.006	0			
**PP**	0.0042	**0.0157**	0.0045	0.0048	0		
**CAS**	0.0019	**0.0191**	0.0024	0.0051	0.0011	0	
**SA**	0.0043	**0.0164**	0.002	0.005	0.0017	0.0006	0

Significant *P* values (< 0.05) after sequential Bonferroni correction are in bold. OUT, outside MPA.

**Fig 2:**
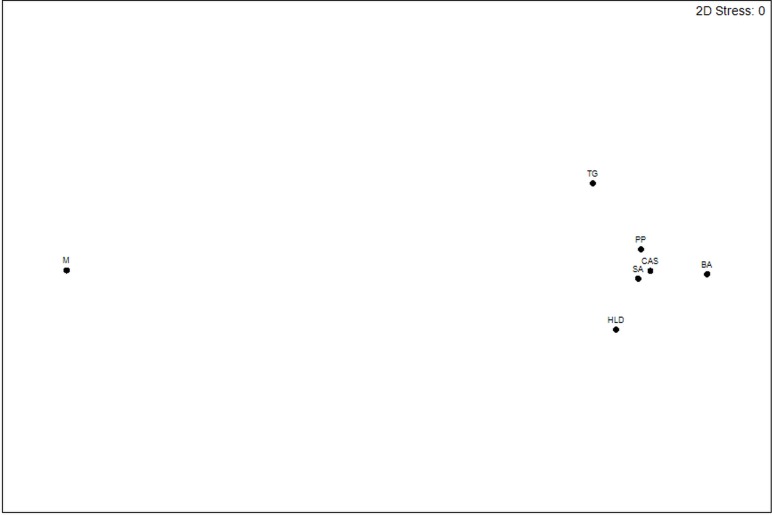
Multidimensional scaling (MDS) plot of pairwise *F*st distances between adult samples. See Fig 1 for legends.

Pairwise *F*st values were significant between Monopoli (M) and all other samples with values ranging from 0.0140 to 0.0256 (Table 1, Fig 2), except for the MPA (TG) sample. Pairwise *F*st values were also significant between Bari (BA) and the MPA (TG) with an *F*st = 0.0084 (Table 1, Fig 2).

The Bayesian analysis “STRUCTURE” revealed no clear genetic structure between adult samples (S1 Fig).

### Juvenile population’s genetic diversity and structure

Allelic richness (Ar) ranged from 13.37 to 15.12. He and Ho ranged from 0.77 to 0.83 and from 0.72 to 0.83, respectively (S2 Table). Significant differences were found for genetic diversity indices among samples using one-way ANOVA (*P* < 0.05) with Torre a Mare (TAM), Punta Penne (PP) and Torre Pozzella (TP) locations showing the lowest values (S2 Table).

The AMOVA analysis showed that only 1% of the genetic variation was explained by genetic differences among the 14 samples with an overall *F*st equal to 0.011 (*P* < 0.001). Levels of differentiation were low but significant in numerous pairwise comparisons once a sequential Bonferroni adjustment was applied (*F*st values ranging from 0.009 to 0.035, S4 Table). The highest significant value of differentiation was found between TP and TAM with *F*st = 0.035. The TAM sample appeared significantly different from all of the other samples except for PP (S4 Table and S2 Fig).

The Bayesian analysis “STRUCTURE” identified 11 clusters amongst the juveniles (based on Evanno’s ΔK) sampled with similar proportions of membership to each cluster with the exception of individuals sampled at site 12 (PP) and 13 (TAM) (S5 Table). These juvenile samples show higher proportion membership to cluster one (i.e. red, S5 Table) than all the others. Juveniles sampled in PP and TAM arise to be genetically different from the rest (S2 Fig).

### Juvenile and adult populations

An AMOVA was used in an analysis of all individuals sampled, for both adults and juveniles, to explore how juveniles may segregate with respect to adults. The analysis showed that 1% of the genetic variation was explained by genetic differences among the pooled individuals with an overall *F*st equal to 0.015 (*P* < 0.001). Levels of differentiation, as measured by pairwise *F*st, were low but significant between most of the samples (values ranging from 0.0077 to 0.057)

The MDS two-dimensional plot showed an overall grouping of all individuals with the exception of juveniles from Punta Penne (PP), Torre a Mare (TAM), and adults from Monopoli (M). Such observations were similar to those previously conducted where juveniles and adults were analyzed separately.

### Assignment tests

A total of 107 juveniles out of 755 (14%) were assigned to one of the adult samples. Most of these juveniles were assigned to Monopoli (M) or Torre Guaceto (TGMPA) adult samples (61 and 21, respectively). Conversely, 62% juveniles were left unassigned because they were assigned to more than one adult sample (Table 2).

**Table 2:** Results of assignment analysis with GENECLASS2. Juveniles were assigned to one of the seven possible adult populations if the likelihood of their genotype occurring in that population was greater than 0.05, when compared to a distribution of 10^4^ simulated genotypes from that population. Juveniles that had a likelihood superior than 0.05 were considered to have being originated from one of the adult populations. If a juvenile was assigned to more than one population with likelihood greater than 0.5 it was left unassigned. Juveniles with likelihood less than 0.05 in all populations were assumed to be immigrants. The results of the test in TGMPA are in bold. See Fig 1 for legends.

Juveniles	Assigned juveniles to adult populations							Immigrants	Unassigned
	Adults								
	OUT			MPA	OUT				
	BA	M	HLD	TGMPA	PP	SA	CAS		
SG (78)	0	5	1	**2**	0	0	1	19	40
TAM (53)	0	7	0	**1**	0	0	0	8	36
PM (53)	0	7	0	**1**	1	0	1	8	35
TI (50)	0	5	0	**1**	1	0	1	8	34
HLD (49)	0	3	1	**2**	1	0	1	10	32
TP (53)	0	3	1	**2**	0	1	0	16	30
**PPG (50)**	**0**	**8**	**1**	**1**	**0**	**0**	**1**	**10**	**29**
**TB (44)**	**0**	**1**	**0**	**2**	**0**	**0**	**0**	**13**	**28**
TR (52)	0	2	0	**2**	0	1	0	17	31
PP (58)	0	3	0	**2**	0	1	0	10	42
CAS (51)	0	5	1	**1**	0	0	2	15	26
TRM (53)	0	3	2	**2**	1	0	1	13	30
SF (56)	0	2	2	**1**	0	0	0	19	32
SA (55)	0	7	0	**1**	0	0	1	13	34
	0	61	9	**21**	4	3	9	179	459

A total of 179 juveniles out of 755 (24%) had a probability lower than 0.05 of belonging to any sample and were designated as being immigrants coming from genetically distinct populations than those of the adults in our study area. Therefore, the remaining 76% of juveniles collected in the 14 sites originated from adults collected in the 7 locations along the 200 km of the study area.

Regarding TGMPA, 24% of juveniles (23 out of 94) are immigrants. Twelve % of the juveniles collected in TGMPA (Table 2) are originated from adults outside TGMPA but within our study area. Whereas, about 3% of juveniles (18 out of 655; Table 2) were collected outside TGMPA and originated inside. On the other hand, 3 juveniles out of 94 collected in TGMPA are originated from adults collected at TGMPA revealing 3% of self-recruitment within TGMPA.

## Discussion

The genetic structure analysis of the two-banded sea bream *Diplodus vulgaris* along ~ 200 km of the Apulian Adriatic coast (SW Adriatic Sea) did not reveal any structured pattern attributable to the presence of TGMPA with respect to distance. However, we found local genetic heterogeneity for juveniles from Punta Penne (PP) and Torre a Mare (TAM); and for adults from Monopoli (M), resulting in genetically differentiated entities. In addition, we demonstrate that 12% of juveniles sampled in TGMPA are originated from adults in the surrounding fished areas, while 3% of juveniles sampled outside TGMPA are originated inside. On the other hand, about 24% of juveniles collected in the 14 sites located along the 200 km of coast originate from outside the surveyed area, where they display genetic combinations that we would not expect to find within the adults sampled from the study area. These results demonstrate patterns of connectivity in relation to the local MPA

### Genetic diversity and population structure

Looking at the quality of the dataset, some of the loci analyzed in this study showed a deviation from expectations for HWE with a significant deficit of heterozygotes in some sampling locations. Such a deficit may be due to various processes such as inbreeding, assortative mating, Wahlund effect [59] or null alleles. Since no homozygozity for null alleles was observed, we excluded the null allele hypothesis. The inbreeding hypothesis can also be excluded because inbreeding should display a deficit across all independent polymorphic loci, a pattern not found in this study. Despite no conclusive evidences are available, it is commonly assumed that *Diplodus* species spawn in group [60] and therefore in this case the assortative mating that is usually influenced by male and female courtship or interaction before release of eggs can be excluded. The Wahlund effect, by which the presence of different genetic pools is merged within a single sample and can cause an excess of homozygotes, is the likely hypothesis.

No differentiation was found in the genetic diversity indices among the adult samples throughout the 200 km range within the South West Adriatic Sea. This result indicates that TGMPA is not an isolated protected location and that there is a patchwork of genetic connectivity among sampled locations. This result is in agreement with the study of Pujolar et al. [61] that showed an overall genetic homogeneity for the congeneric *Diplodus sargus*. The genetic similarity indicates that at the scale of this study, gene flow is sufficient to maintain homogeneity in allele frequencies between TGMPA and the surrounding fished grounds along the sampling area (i.e. 200 km). The observed genetic similarity results from the recent isolation of the Adriatic population following the last sea level rise and continuous dispersion by sea currents dominating the western Adriatic [62] during the *D*. *vulgaris* spawning period (i.e. mainly winter) and the exchanges and potentially extensive dispersal at juvenile stage [63].

Overall, little evidence of genetic structure was found among the 7 adult samples. Only the adults sampled in Monopoli appeared genetically different from all the others (except samples of TGMPA). In the meantime Monopoli samples show the lowest values of genetic diversity indices (Ar = 14.26, Ho = 0.71 and He = 0.81). The isolate genetic divergence of Monopoli samples suggest for some local process often enrolled into the concept of chaotic genetic patchiness. The temporal variability in the sampling could be a possible explanation for this divergence as the sampling occurred between 2010 and 2011 for all locations except for Monopoli which occurred entirely in 2011. The differentiation of the Monopoli population could be due to post-settlement natural selection events [64] since differentiation may occur over relatively fine spatial scales even if there is considerable gene flow when selection is strong [65, 66]. Finally, it is difficult to conclude which processes drive the genetic divergence of the Monopoli adult sample, though the confirmation of genetic heterogeneity suggests variability in genetic supply or/and post-settlement processes.

Overall, high values of genetic diversity and little evidence of genetic structure were found for juvenile samples. These results are most likely due to significant connectivity and admixture among the 14 sampling sites collected within a fairly limited spatial scale (maximum of 200 km apart) as well as habitat continuity along the sampling area. These findings are also consistent with the findings by Di Franco et al. [63] where extensive dispersal during the juvenile stage of the *D*. *vulgaris* of up to 165 km was reported. These authors also highlighted the existence of three major natal sources of propagules responsible for the replenishment of their study area (i.e. 180 km of coastline), suggesting that propagule dispersal extends to at least 90 km [63].

Some of the juvenile sites (e.g. TAM and PP) appeared significantly different from others although *F*st values were low (i.e. as expected in such regional survey). The pairwise *F*st analysis and the Bayesian method both demonstrated that some juveniles sampled in TAM and PP were genetically different from all other samples. TAM and PP samples showed the lowest genetic diversity indices (Ar = 13.37, Ho = 0.77, and Ar = 13.81, Ho = 0.79 respectively) among all samples. This observed differentiation most likely originates from genetic drift linked to a reproduction event involving a limited number of adults. In fact, the localized heterogeneity found in the genetic constitution of some juveniles sampled could be interpreted as the result of a large variance in the local reproductive success [67]. A high variance in reproductive success, implying low effective number of genitors at the origin of a cohort, will favor the occurrence of genetically distinct pools of juveniles at small geographic or temporal scales [68]. In addition, the chaotic genetic patchiness [69–71] observed here could be the result of the occurrence of turbulence in flow that would lead to patchiness in larval dispersal even along straight coastlines [72]. This is in agreement with the findings from Di Franco et al. [32] which suggest the presence of larval patches for this species along this study area based on spatial heterogeneity of larval traits. In this case, coastal eddies might have swept larvae together into ‘patches’ from the reproduction of a small number of adults that were then transported to settlement sites with limited mixing possibilities, rather than becoming an homogeneous larval pool. The resulting patterns of connectivity among sites incorporate high levels of asymmetry and stochasticity [73, 74], that could have contributed to the apparent chaotic genetic patchiness found here. It is worth noting that recruitment is a spatially and temporally stochastic process and what is observed at one site might represent only a single recruited cohort, with other distinct cohorts settling at a neighbouring site [68].

The lack of high genetic structure across the study area reflects the presence of significant gene flow within the studied distribution range. Conversely, and within the context of global homogeneity, the genetic patchiness found at some sites and locations reflects temporal variation in recruitment and variance of local reproductive success.

### Assignment tests

The assignment method performed by GeneClass2 has the advantage taking into account the possibility of not having sampled all potential populations [58]. Using this method, we found that 24% of juveniles cannot be assigned to the local population, where they display genetic combinations that we would not expect to find within the adults collected from the range of our sampling. It is very difficult to define the origin of these immigrants since the oceanography during the *D*. *vulgaris’* spawning period (i.e. mainly winter) is dominated by a multitude of turbulence and gyres at different spatial scales [62]. Therefore the remaining 76% are self-recruitment from the local population, of which we cannot identify the boundaries from our study because we did not sample large enough. About 62% of the juveniles were left unassigned since the validity of the assignment method is sensitive to the level of population differentiation that was very weak in our case [75]. On the other hand, a small number of juveniles (14%) were assigned to only one of the adult samples (107 juveniles out of 755). A decreasing density gradient of assigned juveniles was detected from north to south. Most of these juveniles were assigned to Monopoli and TGMPA (57% and 19.6%, respectively) that also appeared as the most divergent locations (adult samples).

Regarding TGMPA, 12% of juveniles sampled inside the MPA are originated from adults in the surrounding fished areas, while 3% of juveniles sampled outside TGMPA are originated inside. In addition, 24% of juveniles sampled in TGMPA were found to be immigrants. Therefore, these results demonstrate patterns of connectivity in relation to the local MPA. The estimation of self-recruitment (3%) in TGMPA based on assignment tests should be treated with caution since low genetic differentiation was found in the *F*st and STRUCTURE results.

## Conclusion

This study showed that TGMPA is part of an interconnected system and that the high gene flow among *Diplodus vulgaris* populations is maintaining the local population supply. Measuring gene flow of *D*. *vulgaris* by genetic markers has allowed us to elucidate the distance of effective larval dispersal of this species in the study area.

The spatial scale of the sampling outside the MPA cannot account for the entire dispersal kernel of individuals produced in the MPA making some limitation of the present work. However, recent paper that provided kernel of larval dispersal [76, 77] demonstrated a rapid diminution of the dispersal, whatever the species and suggesting that long dispersal event are not common.

Estimates of larval dispersal distances can subsequently be used to optimize MPA placing within a network since an MPA has been proposed in the area of Otranto-Tricase (50 km south of San Andrea (SA)). This new MPA may improve the potential connectivity between TGMPA in the Adriatic and in Porto Cesareo MPA in the Ionian Sea. Thus, the results may serve as a baseline for future studies looking into the design and implementation of a potential network between TGMPA, Otranto-Tricase and Porto Cesareo MPAs.

## Supporting Information

S1 Table Summary of the genetic variation for eight microsatellites loci of adults sampled in 7 locations. (PDF)

S2 Table Summary of the genetic variation for eight microsatellites loci of juveniles sampled in 14 sites. (PDF)

S3 Table Description of *D*. *vulgaris* microsatellite loci. The original primers were developed in Roques et al. [44, 45].
(PDF)

S4 Table Matrix of pairwise *F*st between juvenile samples. See Fig 1 for legends.
(PDF)

S5 Table Values of ΔK calculated as in Evanno et al. [56] and based on the log likelihood of the data given by STRUCTURE 2.2.3 for each number of clusters assumed. **Barplot graphical display of STRUCTURE analysis for K = 11 clusters based on Evanno’s ΔK.** Each juvenile individual was represented by a vertical bar. Each color represents the relative membership proportion of each juvenile to each of the 11 clusters.
(PDF)

S6 Table Matrix of pairwise Dst between adult samples. (PDF)

S7 Table Matrix of pairwise Dst between juvenile samples. (PDF)

S1 Fig Barplot graphical display of STRUCTURE analysis for K = 3 clusters based on Evanno’s ΔK. Each adult individual was represented by a vertical bar. Each color represents the relative membership proportion of each adult to each of the 3 clusters.
(TIF)

S2 Fig Multi dimensional scaling plot of pairwise *F*st distances between juvenile samples. See Fig 1 for legends.
(TIF)
