# Supplementary material for: Patterns of Fish Connectivity between a Marine Protected Area and Surrounding Fished Areas
Source: PLoS One. 2016 Dec 1;11(12):e0167441. doi: 10.1371/journal.pone.0167441 (PMC5131959; doi:10.1371/journal.pone.0167441)
Supplement: S1 Table — (PDF) [file pone.0167441.s001.pdf]

S1 Table. Summary of the genetic variation for eight microsatellites loci of adults sampled in 7 locations. Ns, number of individual sampled; Na, number of alleles; Ho and He, observed and expected heterozygosity respectively, *F*is, inbreeding coefficient; HWE, Hardy-Weinberg equilibrium, Ar, allelic richness. OUT, outside MPA; n.s: not significant results, * significant departure from HWE (*P* < 0.05).

**Sampling locations**		**Bld15**	**Dv11**	**Om27**	**Om38**	**Dv33**	**Dv6**	**Dv61**	**Dv4**
OUT Ns = 86 **BA**	Na	23	6	22	14	13	13	31	16
	Ho	0.78	0.53	0.72	0.52	0.790	0.84	0.73	0.77
	He	0.91	0.52	0.89	0.60	0.88	0.77	0.95	0.90
	*F*is	0.15	-0.02	0.20	0.14	0.10	-0.10	0.23	0.14
	HWE *P*-value	0*	0*	0*	0*	0.07n.s	0*	0*	0*
	Ar					14.40			
	Mean Ho					0.71			
	Mean He					0.81			
OUT Ns = 42 **M**	Na	16	8	18	15	11	13	24	12
	Ho	0.76	0.24	0.89	0.65	0.69	0.75	0.75	0.92
	He	0.91	0.41	0.90	0.79	0.80	0.82	0.93	0.85
	*F*is	0.16	0.41	0	0.18	0.14	0.09	0.19	-0.09
	HWE *P*-value	0.04n.s	0*	0.26n.s	0n.s	0.13n.s	0*	0*	0.95n.s
	Ar					14.26			
	Mean Ho					0.71			
	Mean He					0.81			
OUT Ns = 82 **HLD**	Na	16	7	19	15	15	16	32	17
	Ho	0.87	0.35	0.80	0.59	0.86	0.86	0.85	0.87
	He	0.88	0.48	0.89	0.62	0.87	0.84	0.95	0.89
	*F*is	0.02	0.28	0.09	0.04	0.01	-0.02	0.18	0.01
	HWE *P*-value	0.82n.s	0*	0*	1n.s	0.75n.s	0.22n.s	0n.s	0*
	Ar					13.9			
	Mean Ho					0.75			
	Mean He					0.81			
Ns = 69	Na	17	6	21	11	18	10	30	17
**TGMPA**	Ho	0.88	0.38	0.78	0.66	0.70	0.94	0.81	0.79

	He	0.88	0.43	0.87	0.66	0.89	0.79	0.95	0.87
	*F*is	0.01	0.11	0.10	-0.01	0.22	-0.19	0.15	0.09
	HWE *P*-value	0.62n.s	0*	0.1n.s	0*	0*	0.62n.s	0n.s	0.75n.s
	Ar					13.99			
	Mean Ho					0.74			
	Mean He					0.79			
OUT Ns = 78 **PP**	Na	19	8	21	13	14	12	29	16
	Ho	0.69	0.34	0.86	0.51	0.7	0.89	0.86	0.79
	He	0.89	0.44	0.91	0.61	0.88	0.81	0.95	0.89
	*F*is	0.22	0.25	0.06	0.16	0.20	-0.09	0.09	0.11
	HWE *P*-value	0*	0n.s	0.39n.s	0*	0*	0.03n.s	0*	0*
	Ar					14.05			
	Mean Ho					0.70			
	Mean He					0.80			
OUT Ns = 85 **CAS**	Na	75	83	85	85	82	85	77	80
	Ho	0.76	0.37	0.8	0.58	0.79	0.85	0.73	0.75
	He	0.88	0.49	0.90	0.61	0.90	0.82	0.94	0.88
	*F*is	0.14	0.24	0.11	0.05	0.12	-0.03	0.23	0.14
	HWE *P*-value	0*	0*	0.04n.s	0*	0*	0.30n.s	0*	0*
	Ar					15.27			
	Mean Ho					0.70			
	Mean He					0.81			
OUT Ns = 83 **SA**	Na	18	8	24	20	16	16	28	18
	Ho	0.82	0.26	0.81	0.7	0.79	0.94	0.68	0.89
	He	0.89	0.45	0.91	0.66	0.89	0.83	0.93	0.88
	*F*is	0.08	0.43	0.11	-0.06	0.11	-0.13	0.27	-0.01
	HWE *P*-value	0.04n.s	0*	0*	0*	0*	0.02n.s	0*	0.01n.s
	Ar					15.05			
	Mean Ho					0.74			
	Mean He					0.81			
