# Supplementary material for: Patterns of Fish Connectivity between a Marine Protected Area and Surrounding Fished Areas
Source: PLoS One. 2016 Dec 1;11(12):e0167441. doi: 10.1371/journal.pone.0167441 (PMC5131959; doi:10.1371/journal.pone.0167441)
Supplement: S2 Table — (PDF) [file pone.0167441.s002.pdf]

S2 Table. Summary of the genetic variation for eight microsatellites loci of juvenile sampled in 14 sites. Ns, number of individual sampled; Na, number of alleles; Ho and He, observed and expected heterozygosity respectively, *F*is, inbreeding coefficient, HWE, Hardy-Weinberg equilibrium, Ar, allelic richness. OUT, outside MPA. n.s: not significant results, * significant departure from HWE ($P < 0.05$).

**Sampling sites**		**Bld15**	**Dv11**	**Om27**	**Om38**	**Dv33**	**Dv6**	**Dv61**	**Dv4**
OUT<br>Ns = 78<br>**SG**	Na	19	5	21	13	16	12	33	15
	Ho	0.81	0.37	0.84	0.59	0.78	0.92	0.71	0.82
	He	0.91	0.47	0.89	0.60	0.88	0.81	0.95	0.88
	*F*is	0.11	0.21	0.06	0.02	0.11	-0.14	0.25	0.06
	HWE *P*-value	0*	0n.s	0*	1n.s	0.01n.s	0*	0*	0.65n.s
	Ar	14.33							
	Mean Ho	0.73							
	Mean He	0.81							
OUT<br>Ns = 53<br>**TAM**	Na	18	6	18	8	14	10	29	17
	Ho	0.87	0.42	0.70	0.75	0.56	0.98	0.89	0.73
	He	0.90	0.57	0.73	0.57	0.73	0.79	0.94	0.89
	*F*is	0.04	0.27	0.04	-0.33	0.24	-0.25	0.06	0.18
	HWE *P*-value	0.23n.s	0*	0*	0*	0n.s	0.1n.s	0.02n.s	0.06n.s
	Ar	13.37							
	Mean Ho	0.73							
	Mean He	0.77							
OUT<br>Ns = 53<br>**PM**	Na	22	5	17	14	13	8	33	15
	Ho	0.94	0.70	0.90	0.68	0.78	0.96	0.83	0.8
	He	0.91	0.57	0.90	0.62	0.87	0.78	0.95	0.88
	*F*is	-0.03	-0.22	-0.01	-0.09	0.11	-0.23	0.13	0.09
	HWE *P*-value	0.37n.s	0.11n.s	0.03n.s	0.97n.s	0.2n.s	0.33n.s	0.01n.s	0.74n.s
	Ar	14.36							
	Mean Ho	0.83							
	Mean He	0.82							

**Sampling sites**		**Bld15**	**Dv11**	**Om27**	**Om38**	**Dv33**	**Dv6**	**Dv61**	**Dv4**
TGMPA<br>Ns = 44<br>**TB**	Na	43	44	38	39	37	43	43	38
	Ho	0.86	0.7	0.79	0.49	0.62	1	0.84	0.82
	He	0.87	0.61	0.89	0.63	0.87	0.77	0.94	0.89
	*F*is	0.01	-0.15	0.11	0.23	0.28	-0.29	0.1	0.09
	HWE *P*-value	0.99n.s	0.12n.s	0*	0*	0.02n.s	0.99n.s	0.44n.s	0.13n.s
	Ar	14.52							
	Mean Ho	0.77							
	Mean He	0.82							
OUT<br>Ns = 52<br>**TRM**	Na	19	4	20	15	15	9	28	17
	Ho	0.80	0.84	0.84	0.63	0.78	0.96	0.92	0.85
	He	0.91	0.59	0.90	0.62	0.90	0.80	0.93	0.90
	*F*is	0.12	-0.42	0.07	-0.02	0.13	-0.2	0.01	0.05
	HWE *P*-value	0.2n.s	0n.s	0.03n.s	1n.s	0.61n.s	0.03n.s	0.99n.s	0.04n.s
	Ar	14.55							
	Mean Ho	0.82							
	Mean He	0.83							
OUT<br>Ns = 58<br>**PP**	Na	20	6	20	10	15	11	28	14
	Ho	0.83	0.40	0.78	0.79	0.67	0.90	0.85	0.76
	He	0.90	0.52	0.79	0.57	0.81	0.82	0.94	0.90
	*F*is	0.08	0.24	0.02	-0.39	0.18	-0.09	0.09	0.15
	HWE *P*-value	0.59n.s	0*	0*	0*	0*	1n.s	0.01n.s	0.89n.s
	Ar	13.81							
	Mean Ho	0.75							
	Mean He	0.79							

	Na	18	6	20	14	13	7	25	16
OUT	Ho	0.84	0.26	0.91	0.65	0.84	0.98	0.78	0.8
Ns = 50	He	0.90	0.41	0.91	0.71	0.88	0.77	0.94	0.87
**TI**	*F*is	0.07	0.36	-0.01	0.09	0.04	-0.27	0.17	0.08
	HWE *P*-value	0.78n.s	0*	0.12n.s	0*	0.49n.s	0.13n.s	0*	0.42n.s
	Ar	14.19							
	Mean Ho	0.76							
	Mean He	0.81							
	Na	20	6	19	12	15	8	26	15
	Ho	0.73	0.47	0.71	0.60	0.77	0.92	0.76	0.80
	He	0.90	0.55	0.92	0.63	0.89	0.76	0.94	0.87
OUT	*F*is	0.19	0.15	0.22	0.04	0.13	-0.21	0.19	0.07
Ns = 49	HWE *P*-value	0*	0.16n.s	0.02n.s	0.91n.s	0.3n.s	0.66n.s	0*	0*
**HLD**	Ar	14.22							
	Mean Ho	0.72							
	Mean He	0.81							
	Na	18	5	19	11	14	10	28	16
	Ho	0.88	0.18	0.86	0.75	0.88	0.96	0.7	0.88
	He	0.89	0.25	0.90	0.75	0.88	0.79	0.94	0.89
OUT	*F*is	0.01	0.29	0.05	0	0	-0.22	0.25	0.01
Ns = 53	HWE *P*-value	0.49n.s	0*	0.19n.s	0*	0.39n.s	0.01n.s	0*	0.7n.s
**TP**	Ar	14.05							
	Mean Ho	0.76							
	Mean He	0.79							
	Na	20	5	20	13	14	10	29	15
	Ho	0.70	0.60	0.87	0.65	0.76	0.96	0.78	0.90
	He	0.91	0.58	0.87	0.66	0.9	0.79	0.95	0.87
TGMPA	*F*is	0.23	-0.03	0	0.02	0.15	-0.21	0.18	-0.03
Ns = 50	HWE *P*-value	0.02n.s	0.07n.s	0.84n.s	0.04n.s	0.47n.s	0.76n.s	0n.s	0.67n.s
**PPG**	Ar	14.66							
	Mean Ho	0.79							
	Mean He	0.83							

	Na	20	5	20	14	17	12	25	19
OUT	Ho	0.92	0.88	0.92	0.67	0.71	0.90	0.80	0.72
Ns = 51	He	0.90	0.62	0.90	0.67	0.89	0.8	0.93	0.86
**CAS**	*F*is	-0.03	-0.42	-0.03	0.01	0.2	-0.13	0.13	0.16
	HWE *P*-value	0.99n.s	0.01n.s	1n.s	0.17n.s	0*	0n.s	0.01n.s	0*
	Ar	15.12							
	Mean Ho	0.82							
	Mean He	0.83							
	Na	17	4	20	13	14	10	30	17
	Ho	0.81	0.43	0.70	0.51	0.70	0.90	0.89	0.88
	He	0.90	0.43	0.91	0.55	0.89	0.79	0.95	0.90
OUT	*F*is	0.1	-0.01	0.23	0.08	0.21	-0.14	0.06	0.02
Ns = 53	HWE *P*-value	0.02n.s	0.32n.s	0.01n.s	0.94n.s	0*	0.76n.s	0.01n.s	0*
**TR**	Ar	14.43							
	Mean Ho	0.73							
	Mean He	0.80							
	Na	19	6	21	13	14	9	30	18
	Ho	0.91	0.79	0.82	0.52	0.74	0.91	0.84	0.80
	He	0.91	0.66	0.9	0.54	0.89	0.78	0.95	0.90
OUT	*F*is	0	-0.2	0.09	0.04	0.16	-0.17	0.12	0.11
Ns = 56	HWE *P*-value	0n.s	0*	0*	1n.s	0.73n.s	0.83n.s	0n.s	0n.s
**SF**	Ar	14.88							
	Mean Ho	0.79							
	Mean He	0.82							
	Na	15	5	19	15	17	9	29	17
	Ho	0.83	0.56	0.89	0.62	0.75	0.91	0.83	0.91
	He	0.90	0.52	0.90	0.58	0.90	0.76	0.94	0.90
	*F*is	0.07	-0.09	0.01	-0.06	0.18	-0.19	0.11	-0.01
OUT	HWE *P*-value	0.03n.s	0*	0.12n.s	0.45n.s	0.11n.s	0.01n.s	0n.s	0.98n.s
Ns = 55	Ar	14.36							
**SA**	Mean Ho	0.78							
	Mean He	0.81							
