# Supplementary material for: Patterns of Fish Connectivity between a Marine Protected Area and Surrounding Fished Areas
Source: PLoS One. 2016 Dec 1;11(12):e0167441. doi: 10.1371/journal.pone.0167441 (PMC5131959; doi:10.1371/journal.pone.0167441)
Supplement: S3 Table — The original primers were developed in Roques et al. [44, 45]. (PDF) [file pone.0167441.s003.pdf]

S3 Table. Description of *D. vulgaris* microsatellite loci. The original primers were developed in Roques et al. (31, 32). 'MOD' in primer names indicates primer sequences redesigned in this study. Ho and He, observed and expected heterozygosity respectively, *F*is, inbreeding coefficient.

Locus	Primer sequences	Nb alleles	Ar	Allele size (bp)	He	Ho	*F*is	References	Acession number
**Om38**	F-AGCCGGCTGAGCTCCATAATAACC	10	11.74	180-240	0.62	0.64	-0.02	Roques *et al.* (2006)	EF064300
	R-TGCCCTCTTGTCACACCAGGTCAC								
**Om27**	F-TTGGCTCATTAGACAAAGGCACAC	14	17.96	280-340	0.88	0.82	0.06	Roques *et al.*(2006)	EF064305
	R-GGGCGCTGAAACAATAGCCGTGTT								
**Dv33**	F-GCCGGGCTCGACATTGACACTGAA	17	13.03	280-360	0.87	0.74	0.15	Roques *et al.* (2007)	EF064291
	R-GCAGCCAGCAGAGCTTAAAGAACT								
**Dv4**	F-GCGGTTATGTATACGTTGCGTTTA	16	14.39	220-300	0.89	0.82	0.07	Roques *et al.* (2007)	EF064292
	R-TTGGCGTTGAACAGAAGTCAGACA								
**Dv11**	F-GGCCCGCTTTATTCTCAGTCTCAA	7	6.00	160-180	0.53	0.54	-0.03	Roques *et al.* (2007)	EF064289
	R-TGCAGGGAACAGAGGGATGACAG								
	**FMOD**-CTTAACTGGGAGCACAGGCCCG								
	**RMOD**-TGTGCAGGGAACAGAGGGATGAC[illegible]								
**Dv61**	F-TGGGGACTCTCAGAATCATCACAA	28	24.89	120-220	0.94	0.82	0.013	Roques *et al.* (2007)	EF064293
	R-TGGAAAAAGCCCTCTGGACAAAAG								
	**FMOD**-GCTTTGCTGGCTTTCTGACT								
	**RMOD**-GCCCTCTGGACAAAAGAACA								
**Dv6**	F-GGGCAAACAGGAGCAAAAAGCCAG	13	11.40	120-240	0.79	0.94	-0.20	Roques *et al.* (2007)	EF064297
	R-AGCCGCAGTTGATTTACAGAGTGT								
	**FMOD**-TACCACAGGACAAACCGACA								
	**RMOD**-GCCCTCTTTTGCCTATGTTG								
**Bld15**	F-CACCAATCACTCGGCTTCAC	18	15.99	160-220	0.90	0.84	0.07	Franch *et al.* (2006)	DQ851279
	R-GCAGCTAAAAGCTACTGGGAGA								
