# Supplementary material for: Patterns of Fish Connectivity between a Marine Protected Area and Surrounding Fished Areas
Source: PLoS One. 2016 Dec 1;11(12):e0167441. doi: 10.1371/journal.pone.0167441 (PMC5131959; doi:10.1371/journal.pone.0167441)
Supplement: S4 Table — See Fig 1 for legends. (PDF) [file pone.0167441.s004.pdf]

	OUT SG	OUT TAM	OUT PM	OUT TI	OUT HLD	OUT TP	TGMPA PPG	TGMPA TB	OUT TR	OUT PP	OUT CAS	OUT TRM	OUT SF	OUT SA
**SG**	0													
**TAM**	**0.0243**	0												
**PM**	0.0065	**0.0104**	0											
**TI**	0.0037	**0.0272**	**0.0116**	0										
**HLD**	0.0016	**0.0293**	0.0021	0.0021	0									
**TP**	0.0056	**0.035**	**0.0168**	-0.0018	0.0059	0								
**PPG**	0.0027	**0.0155**	0.0007	0.0064	0.0017	**0.0116**	0							
**TB**	0.0091	**0.0151**	-0.0009	**0.0187**	0.0063	**0.0206**	-0.0007	0						
**TR**	-0.002	**0.0305**	0.0064	0.0034	-0.0006	0.0038	0.0061	**0.0122**	0					
**PP**	**0.0118**	0.0046	0.0058	**0.0121**	**0.0199**	**0.0199**	0.0055	**0.015**	**0.015**	0				
**CAS**	**0.0094**	**0.0267**	0.0022	**0.0173**	0.0078	**0.0233**	-0.0005	-0.0037	0.0075	**0.0172**	0			
**TRM**	**0.009**	**0.0199**	0.0009	**0.0153**	0.0080	**0.0229**	-0.0024	-0.0027	0.0104	**0.0113**	-0.0002	0		
**SF**	**0.0103**	0.0029	0.0019	**0.0183**	0.0056	**0.0261**	0.0035	-0.0003	**0.0109**	**0.0152**	0.002	0.0029	0	
**SA**	**0.0087**	0.0025	-0.0008	**0.0171**	0.0099	**0.0217**	0.0055	-0.0023	0.006	**0.0157**	0.0024	0.0025	0.0055	0

Significant P values (< 0.05) after sequential Bonferroni correction are in bold. OUT, outside MPA.
