# Supplementary material for: Patterns of Fish Connectivity between a Marine Protected Area and Surrounding Fished Areas
Source: PLoS One. 2016 Dec 1;11(12):e0167441. doi: 10.1371/journal.pone.0167441 (PMC5131959; doi:10.1371/journal.pone.0167441)
Supplement: S5 Table — Barplot graphical display of STRUCTURE analysis for K = 11 clusters based on Evanno’s ΔK. Each juvenile individual was represented by a vertical bar. Each color represents the relative membership proportion of each juvenile to each of the 11 clusters. (PDF) [file pone.0167441.s005.pdf]

	OUT	OUT	OUT	MPA	OUT	OUT	OUT
	**BA**	**M**	**HLD**	**TGMPA**	**PP**	**CAS**	**SA**
**BA**	0.0000						
**M**	**0.0812**	0.0000					
**HLD**	**0.0141**	**0.0408**	0				
**TGMPA**	**0.0332**	**0.0462**	**0.0190**	0			
**PP**	**0.0123**	**0.0550**	**0.0152**	**0.0361**	0		
**CAS**	0.0068	**0.0660**	**0.0092**	**0.0178**	**0.0204**	0	
**SA**	**0.0162**	**0.0623**	0.0067	**0.0274**	**0.0126**	0.0053	0

Significant *P* values (< 0.05) are in bold. OUT, outside MPA.
