# Supplementary figures and images for: Patterns of Fish Connectivity between a Marine Protected Area and Surrounding Fished Areas
Source: PLoS One. 2016 Dec 1;11(12):e0167441. doi: 10.1371/journal.pone.0167441 (PMC5131959; doi:10.1371/journal.pone.0167441)

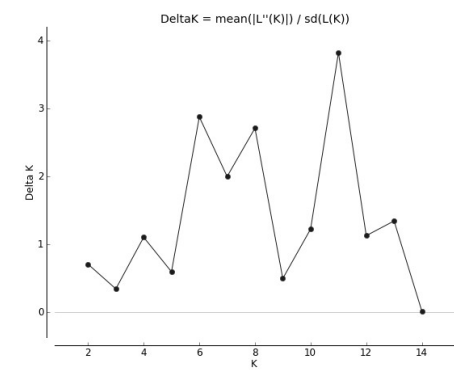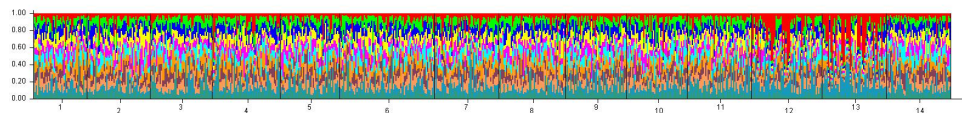

Supplement: S6 Table — (PDF) [file pone.0167441.s006.pdf]

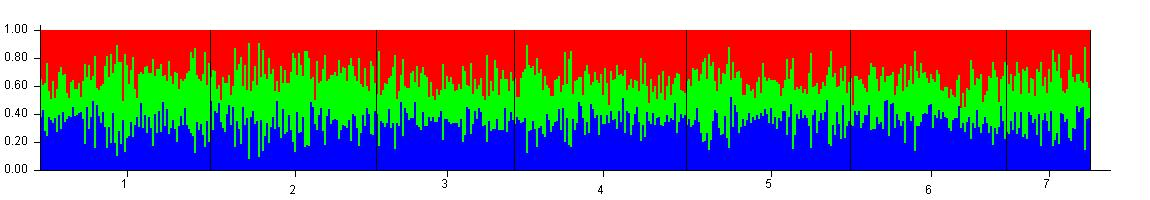

Supplement: S1 Fig — Each adult individual was represented by a vertical bar. Each color represents the relative membership proportion of each adult to each of the 3 clusters. (TIF) [file pone.0167441.s008.tif]

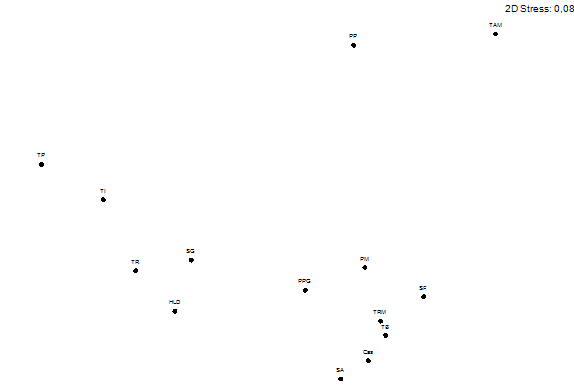

Supplement: S2 Fig — See Fig 1 for legends. (TIF) [file pone.0167441.s009.tif]
