# Supplementary material for: Patterns of Fish Connectivity between a Marine Protected Area and Surrounding Fished Areas
Source: PLoS One. 2016 Dec 1;11(12):e0167441. doi: 10.1371/journal.pone.0167441 (PMC5131959; doi:10.1371/journal.pone.0167441)
Supplement: S7 Table — (PDF) [file pone.0167441.s007.pdf]

	**OUT SG**	**OUT TAM**	**OUT PM**	**OUT TI**	**OUT HLD**	**OUT TP**	**TGMPA PPG**	**TGMPA TB**	**OUT TRM**	**OUT PP**	**OUT CAS**	**OUT TR**	**OUT SF**	**OUT SA**
**SG**	**0**													
**TAM**	**0.1209**	0												
**PM**	**0.0255**	**0.0704**	0											
**TI**	0.0096	**0.1148**	**0.0353**	0										
**HLD**	0.0043	**0.1318**	**0.0292**	0.0110	0									
**TP**	**0.0235**	**0.1572**	**0.0596**	0.0010	**0.0230**	0								
**PPG**	0.0060	**0.0904**	0.0111	**0.0242**	0.0074	**0.0493**	0							
**TB**	**0.0303**	**0.0943**	**0.0217**	**0.0532**	**0.0251**	**0.0826**	0.0040	0						
**TRM**	0.0021	**0.1095**	**0.0336**	**0.0512**	**0.0288**	**0.0778**	0.0075	0.0067	0					
**PP**	**0.0630**	0.0133	**0.0352**	**0.0577**	**0.0778**	**0.0897**	**0.0394**	**0.0782**	**0.0680**	0				
**CAS**	**0.0452**	**0.1463**	**0.0242**	**0.0583**	**0.0443**	**0.0819**	0.0135	0.0019	0.0080	**0.1020**	0			
**TR**	0.0021	**0.1204**	**0.0336**	**0.0171**	0.0113	**0.0262**	**0.0239**	**0.0405**	**0.0362**	**0.0605**	**0.0432**	0		
**SF**	**0.0317**	**0.0807**	**0.0140**	**0.0560**	**0.0198**	**0.0957**	**0.0168**	0.0014	0.0080	**0.0686**	**0.0432**	**0.0355**	0	
**SA**	**0.0222**	**0.1170**	0.0015	**0.0491**	**0.0295**	**0.0677**	0.0118	0.0014	0.0062	**0.0798**	**0.0150**	**0.0225**	**0.0185**	0
